# Long-term outcome of heart failure patients with improved ejection fraction: Results from a real-world registry

**DOI:** 10.1016/j.ijcha.2026.102008

**Published:** 2026-09-15

**Authors:** Yuxiang Luo, Yusuf Z. Sener, Hongtao Tie, Kevin M. Veen, Mattie J. Lenzen, Alina A. Constantinescu, Olivier C. Manintveld, Robert M.A. van der Boon, Wouter C. Meijers, Dominic A. Theuns, Osama Soliman, Kadir Caliskan

**Affiliations:** a Thoraxcenter, Department of Cardiology, Cardiovascular Institute, Erasmus University Medical Center, Rotterdam, the Netherlands; b Department of Cardiothoracic Surgery, The First Affiliated Hospital of Chongqing Medical University, Chongqing, China; c Department of Cardiothoracic Surgery, Erasmus University Medical Center, Rotterdam, the Netherlands; d Royal College of Surgeons in Ireland (RCSI), University of Medicine and Health Sciences, Dublin, Ireland; e Precision Cardiovascular Medicine & Innovation Institute, Cardiovascular Research Institute, Mater Private Network, Dublin, Ireland

**Keywords:** Heart failure, Improved ejection fraction, Outcome, Pharmacotherapy

## Abstract

**Aims:**

This real-world cohort study aims to elucidate the characteristics and long-term outcome of heart failure patients with improved ejection fraction (HFimpEF).

**Methods:**

Data from a prospective observational single-center registry were analyzed. Adult patients with reduced left ventricular ejection fraction (LVEF)(≤40%) were classified as HFimpEF if follow-up LVEF improved to >40%. Primary outcome was defined as the composite of all-cause death, left ventricular assist device implantation, or heart transplantation.

**Results:**

We identified 207 HFimpEF patients (33%) among 635 HF patients with reduced LVEF. Their LVEF improved from 27% (interquartile range, IQR 21,34) to 45% (IQR 43,50, *P* < 0.001) over a median duration of 3.1 years. The 10-year primary outcome-free survival probability was 82.8%. Among the twenty-two observed deaths, only four were attributed to cardiovascular causes. In the multivariable Cox regression analysis, history of cancer (adjusted hazard ratio, aHR 2.72 [95% confidence interval, CI 1.11–6.65], *P* = 0.028) and septal E/e’ ratio (aHR 1.09 [95%CI 1.03–1.16], *P* = 0.006) were identified as independent predictors for primary outcome. A subgroup of patients treated with renin-angiotensin system inhibitors (RASi) and β-blockers alone (*n* = 47) had a higher 10-year survival than those who required additional drugs (94.3%*vs.*78.3%, log-rank *P* = 0.021).

**Conclusions:**

The cardiovascular long-term outcome of HFimpEF patients was generally favorable, with most deaths due to non-cardiovascular causes. A history of cancer and an elevated E/e’ ratio independently predicted the composite of death and advanced HF therapy. Future trials are warranted to determine the optimal long-term pharmacotherapeutic management of this population.

## 1 Introduction

With the advancements in management strategies and therapeutic approaches, the prognosis of patients with heart failure (HF) has significantly improved [1]. Following the introduction of guideline-recommended medical therapy (GRMT), a subset of patients experience considerable improvement in left ventricular ejection fraction (LVEF), reaching a state of asymptomatic left ventricular dysfunction [2]. Currently, these patients are classified as heart failure with improved ejection fraction (HFimpEF), a HF phenotype characterized by a baseline reduced ejection fraction (HFrEF) and subsequent LVEF improvement following GRMT [3]. Although the criteria for defining LVEF improvement vary across clinical guidelines, a follow-up LVEF of at least 40% on echocardiography is generally required [4], [5], [6]. Due to heterogeneity in the definitions of LVEF improvement and variations in therapeutic strategies, the proportion of HFrEF patients who transition to HFimpEF during follow-up differs substantially across studies, ranging from 9% to 60% [7], [8], [9]. Several previous studies have reported the long-term survival outcomes of patients with HFimpEF; however, characterization of their long-term longitudinal trajectories has largely been limited to LVEF, without extension to other biomarkers and echocardiographic parameters [10], [11], [12], [13]. Given the current scarcity of high-quality evidence, there is a lack of guideline recommendations for the long-term clinical management of this population [3], [14]. In this context, we conducted this real-world cohort study, aiming to comprehensively characterize patients with HFimpEF, with a focus on their long-term outcomes and longitudinal clinical trajectories across multiple parameters over a 10-year period in routine practice. In addition, we performed two secondary subgroup analyses comparing outcomes according to pharmacotherapy (renin-angiotensin system inhibitors [RASi] and β-blockers alone *vs.* additional HF medications) and the magnitude of LVEF improvement (<10% *vs.* ≥10%) to provide further insights into the clinical management of HFimpEF.

## 2 Methods

### 2.1 Study population

The patient cohort in this study was derived from the Rijnmond HF registry, a prospective, observational, regional registry that captures HF and cardiomyopathy patients from Erasmus University Medical Center, a tertiary referral center located in Rotterdam, the Netherlands. The registry has been approved by the ethics committee of Erasmus University Medical Center (MEC-2014-100), and conducted in accordance with the Helsinki Declaration, with all patients providing informed consent prior to participation. To ensure broader patient inclusion, we adopted HFimpEF definition proposed by the American College of Cardiology/American Heart Association [4]. Patients were included if they were aged ≥18 years, had a prior diagnosis of HFrEF (≤40%), and demonstrated a follow-up LVEF >40% on echocardiography. Patients with a follow-up duration shorter than six months were excluded. The diagnosis of HFrEF was in alignment with the preceding and contemporary guidelines for HF management [5], [15]. Standardized GRMT was initiated in all patients once they were diagnosed with HFrEF at our institute. This typically included RASi, β-blockers, and mineralocorticoid receptor antagonists (MRA), with loop diuretics and digoxin prescribed as clinically indicated [5]. Given the inclusion period (2014–2021), the use of sodium-glucose cotransporter-2 inhibitors (SGLT-2i) was limited and unrecorded in the registry. During follow-up, medications were adjusted as needed; however, RASi and β-blockers were advised to be consistently prescribed to all patients based on our institutional protocol.

### 2.2 Data collection

Data were prospectively collected with the latest data collection completed in January 2025. Patient characteristics were retrieved at both the time of initial HF diagnosis and the time of LVEF improvement, which included demographic features, medical history, clinical parameters, medication use, laboratory measurements, and imaging data. The late gadolinium enhancement pattern was assessed using cardiac magnetic resonance (CMR), whereas all other imaging parameters were measured by echocardiography. Patients who registered in the Rijnmond HF registry are typically followed up at least annually in our institute, regardless of LVEF level. Follow-up assessment includes evaluation of New York Heart Association (NYHA) functional class, laboratory measurements, and echocardiographic evaluation, with medication adjustments made accordingly. Available recorded data from the follow-up visits were collected. In addition, we recorded the cause of death and contributing factors to death among enrolled HFimpEF patients who died during follow-up.

### 2.3 Outcome measures

The primary outcome was defined as the composite of all-cause death, left ventricular assist device (LVAD) implantation, or heart transplantation, with follow-up initiated at the time of LVEF improvement [12]. For the comparison between HFimpEF patients (target population) and non-HFimpEF patients (population not included in the primary analysis), follow-up was instead initiated at the time of HF diagnosis because no corresponding time point of LVEF improvement was available for the latter group. Secondary outcomes included HF-related hospitalization, new-onset episodes of myocardial infarction, atrial fibrillation, life-threatening ventricular arrhythmias, cerebrovascular events, and cardiac implantable electronic devices (CIED) implantation. HF-related hospitalization and cardiovascular death were defined following clinical trial guidelines [16].

### 2.4 Subgroup analyses

In this section, HFimpEF patients were stratified according to pharmacotherapy and the magnitude of LVEF improvement for comparison, respectively. For the pharmacotherapy-based analysis, the RASi+β-blocker subgroup comprised patients who were dependent on RASi and β-blockers alone during follow-up, whereas the RASi+β-blocker+others subgroup comprised patients who remained on RASi and β-blockers but were additionally prescribed at least one other drug during follow-up, including MRA, loop diuretics, and digoxin. Primary and secondary outcomes were compared between these two subgroups. Furthermore, patients were stratified according to the absolute increase in LVEF observed at the first documented improvement (<10% *vs.* ≥10%). The primary outcome and subsequent longitudinal LVEF trajectories were compared between the two subgroups.

### 2.5 Statistical analysis

Categorical variables were described as frequency (percentage) and compared using Fisher's exact test. Continuous variables were described as median with interquartile range (IQR) and compared using Mann-Whitney *U* test or Kruskal-Wallis test to ensure consistent reporting and robust comparisons given the modest sample size and subgroup sizes. Intra-individual paired comparisons between the time of HF diagnosis and the time of LVEF improvement were conducted using Wilcoxon signed-rank test, McNemar's test, or Stuart-Maxwell test, as appropriate. Competing-risk analysis was conducted using the cumulative incidence function to estimate cause-specific cumulative incidence within the primary outcome, including cardiovascular death, non-cardiovascular death, and LVAD implantation/heart transplantation. HF-related hospitalization was analyzed using the same approach, with the primary outcome treated as a competing event. When analyzing the primary outcome as a whole, Kaplan-Meier method was used, with subgroup comparisons performed *via* log-rank test. Longitudinal trajectories of clinical parameters (including NYHA class, key laboratory findings, and echocardiographic measurements) were estimated and visualized over 10 years according to primary outcome status (event *vs.* no event) using a linear mixed-effects model [17]. Time was modeled with natural splines to allow for non-linear trajectories, and an interaction between time and primary outcome status was included to assess differences in temporal patterns between patients who experienced the primary outcome and those who did not. Patient-level random effects were incorporated to account for within-subject correlation from repeated measurements. The overall between-group difference was assessed using a likelihood ratio test from the linear mixed-effects models. Cox regression analyses were performed to identify predictors of the primary outcome and factors associated with dual-drug therapy [18]. For these analyses, the date of LVEF improvement was defined as time zero, and all variables were assessed at that time point. Univariable regression analyses were initially conducted, and variables with statistical significance were further selected and entered into the multivariable regression through the least absolute shrinkage and selection operator (LASSO) regression [19]. To increase eligible variables in the Cox regression analysis, multiple imputations were performed on variables with less than 20% missing data [20]. An additional Cox regression analysis was performed to evaluate the impact of LVEF improvement on primary outcome, with HF state modeled as a time-dependent covariate and the date of HF diagnosis defined as time zero. Patients who subsequently developed HFimpEF contributed person-time to the HFrEF state before the date of LVEF improvement and to the HFimpEF state thereafter; patients without improvement contributed all person-time to the HFrEF state. All analyses were conducted using R software (Version 4.4.2, R Foundation for Statistical Computing, Vienna, Austria). Values of *P* < 0.050 were considered statistically significant.

## 3 Results

### 3.1 Patient characteristics

Between January 2014 and September 2021, 797 patients were enrolled in the Rijnmond HF registry, among whom 635 were diagnosed with HFrEF. Of these patients, 207 (33%) experienced LVEF improvement (>40%) since diagnosis. Compared with patients in whom LVEF improvement was not observed (*n* = 428), HFimpEF patients were less frequently male (57% *vs.* 72%, *P* < 0.001) and less likely to have an ischemic etiology (13% *vs.* 40%, *P* < 0.001; **Supplementary Table 1**). The patient characteristics at the time of HF diagnosis and at LVEF improvement of HFimpEF patients are shown in Table 1. Over a median follow-up duration of 3.1 years (IQR 1.2,6.6) since HF diagnosis, LVEF improved from 27% (IQR 21,34) to 45% (IQR 43,50, *P* < 0.001), corresponding to a median individual LVEF increase of 19% (IQR 13,26). From HF diagnosis to LVEF improvement, the prevalence of atrial fibrillation (15% *vs.* 21%, *P* < 0.001) and the proportion of patients with CIED implantation (11% *vs.* 55%, *P* < 0.001) increased significantly. At the time of HF diagnosis, 71% of patients were classified as NYHA class III/IV; in contrast, at LVEF improvement, 95% of patients were classified as NYHA class I/II (*P* < 0.001). Meanwhile, the median heart rate decreased from 80 bpm (IQR 67,96) to 66 bpm (IQR 60,75, *P* < 0.001). With regard to RASi use, a significant proportion of patients transitioned from angiotensin-converting enzyme inhibitors (ACEi) to angiotensin II receptor blockers (ARB) or angiotensin receptor-neprilysin inhibitors (ARNi)(*P* < 0.001). Both RASi and β-blockers were consistently administered to all patients. Significant improvement was also observed in laboratory parameters, along with marked evidence of cardiac reverse remodeling on echocardiography, particularly in N-terminal pro-b-type natriuretic peptide (NT-proBNP) levels (188 pmol/L [IQR 74,374] *vs.* 23 pmol/L [IQR 11,51], *P* < 0.001) and the septal E/e’ ratio (13.1 [IQR 10.0,17.8] *vs.* 9.3 [IQR 7.6,11.5], *P* < 0.001).

**Table 1:** Patient characteristics at heart failure diagnosis and at LVEF improvement.

	Total population (*N* = 207)^a^		*P* value
	At heart failure diagnosis	At LVEF improvement	
**Demographics**			
**Male**	118 (57)		
**Age, y**	49 (37, 59)	53 (43, 64)	**<0.001**
**Body mass index, kg/m**^**2**^**(*n*** **=** **202)**	25.1 (22.7, 28.7)	26.2 (23.5, 30.1)	**<0.001**
**Race**			
Caucasian	184 (89)		
African	9 (4)		
Asian	6 (3)		
Other	8 (4)		
**Etiology**			
Ischemic	26 (13)		
Dilated	105 (51)		
Hypertensive	9 (4)		
Noncompaction	26 (13)		
Myocarditis	9 (4)		
Toxic	13 (6)		
Peripartum	3 (1)		
Other	16 (8)		
**Medical history**			
**Hypertension**	48 (23)	52 (25)	0.134
**Atrial fibrillation**	31 (15)	44 (21)	**<0.001**
**Cerebrovascular events**			0.223
Cerebrovascular accident	11 (5)	13 (6)	
Transient ischemic attack	10 (5)	11 (5)	
**Diabetes mellitus**	16 (8)	21 (10)	0.074
**Chronic obstructive pulmonary disease**	12 (6)	16 (8)	0.134
**Obstructive sleep apnea syndrome**	9 (4)	11 (5)	0.480
**Chronic kidney disease**	45 (22)	51 (25)	0.417
**Cancer**	22 (11)	25 (12)	0.248
**CIED implantation**			**<0.001**
Pacemakers	3 (1)	4 (2)	
ICD	11 (5)	69 (33)	
CRT-D	10 (5)	41 (20)	
**Clinical parameters**			
**Duration from diagnosis to improvement, y**	3.1 (1.2, 6.6)		
**NYHA class (*n*** **=** **181)**			**<0.001**
I	3 (2)	111 (61)	
II	50 (28)	61 (34)	
III	85 (47)	9 (5)	
IV	43 (24)	0	
**Heart rate, bpm (*n*** **=** **205)**	80 (67, 96)	66 (60, 75)	**<0.001**
**Systolic blood pressure, mmHg (*n*** **=** **203)**	115 (102,133)	120 (110, 136)	**0.005**
**Diastolic blood pressure, mmHg (n** **=** **203)**	73 (65, 80)	71 (70, 80)	0.268
**QRS duration, ms (n** **=** **203)**	107 (98, 146)	111 (99, 145)	0.088
**Medication use**			
**RASi**			**<0.001**
ACEi	160 (77)	139 (67)	
ARB	27 (13)	35 (17)	
ARNi	20 (10)	33 (16)	
**β-blockers**	207 (100)	207 (100)	1.000
**MRA**	134 (65)	138 (67)	0.665
**Loop diuretics**	158 (76)	148 (71)	0.112
**Digoxin**	77 (37)	81 (39)	0.596
**Laboratory parameters**			
**Glucose, mmol/L (*n*** **=** **184)**	5.8 (5.1, 7.2)	5.7 (5.1, 6.6)	0.115
**eGFR, mL/min/1.73m**^**2**^**(*n*** **=** **193)**	79 (61, 100)	76 (58, 94)	**0.001**
**Urea, mmol/L (*n*** **=** **196)**	6.8 (5.3, 9.3)	6.6 (5.0, 8.3)	0.231
**Sodium, mmol/L (*n*** **=** **198)**	140 (138, 142)	141 (139, 142)	**<0.001**
**Potassium, mmol/L (n** **=** **198)**	4.4 (4.0, 4.6)	4.4 (4.1, 4.7)	0.181
**Total bilirubin, μmol/L (*n*** **=** **136)**	10 (7, 16)	8 (6, 10)	**<0.001**
**AST, U/L (*n*** **=** **189)**	28 (22, 45)	24 (20,31)	**<0.001**
**ALT, U/L (n** **=** **189)**	34 (20, 61)	25 (18, 35)	**<0.001**
**NT-proBNP, pmol/L (*n*** **=** **163)**	188 (74, 374)	23 (11, 51)	**<0.001**
**Hemoglobin, mmol/L (*n*** **=** **196)**	8.7 (7.9, 9.5)	8.6 (7.9, 9.2)	0.931
**Imaging parameters**			
**LA diameter, mm (*n*** **=** **179)**	42 (38, 47)	40 (36, 44)	**<0.001**
**LVEDD, mm (*n*** **=** **193)**	62 (56, 68)	55 (51, 61)	**<0.001**
**LVESD, mm (*n*** **=** **64)**	53 (48, 61)	40 (35, 45)	**<0.001**
**LVEF, %**	27 (21, 34)	45 (43, 50)	**<0.001**
**VCI diameter, mm (*n*** **=** **159)**	17 (13,20)	16 (14, 19)	0.261
**TAPSE, mm (*n*** **=** **119)**	19 (17, 23)	22 (18, 26)	**<0.001**
**E/e’, ratio (*n*** **=** **129)**	13.1 (10.0, 17.8)	9.3 (7.6, 11.5)	**<0.001**
**TR pressure gradient, mmHg (*n*** **=** **82)**	24 (19, 33)	23 (19, 25)	**0.004**
**RV dysfunction (*n*** **=** **117)**	65 (56)	22 (19)	**<0.001**
**LGE pattern in CMR (*n*** **=** **67)**			
None	38 (57)		
Subendocardial	3 (5)		
Midmyocardial	21 (31)		
Transmural	5 (7)		

Data are median (interquartile range) or frequency (percentage). Available cases of variables with missing values are displayed in the row names.

ACEi, angiotensin-converting enzyme inhibitors; ALT, alanine aminotransferase; ARB, angiotensin II receptor blockers; ARNi, angiotensin receptor-neprilysin inhibitors; AST, aspartate aminotransferase; CIED, cardiac implantable electronic devices; CMR, cardiac magnetic resonance; CRT—D, cardiac resynchronization therapy with defibrillators; eGFR, estimated glomerular filtration rate; ICD, implantable cardioverter defibrillators; LA, left atrium; LGE, late gadolinium enhancement; LVEDD, left ventricular end-diastolic diameter; LVEF, left ventricular ejection fraction; LVESD, left ventricular end-systolic diameter; MRA, mineralocorticoid receptor antagonists; NT-proBNP, N-terminal pro-b-type natriuretic peptide; NYHA, New York Heart Association; RASi, renin-angiotensin system inhibitors; RV, right ventricle; TAPSE, tricuspid annular plane systolic excursion; TR, tricuspid regurgitation; VCI, vena cava inferior.

a For each variable, only patients with available data at both heart failure diagnosis and at the time of LVEF improvement were included in the paired comparisons.

### 3.2 The impact of LVEF improvement

Patients with HFimpEF had a higher 10-year primary outcome-free survival probability from the time of HF diagnosis than those in whom LVEF improvement was not observed (91.9% *vs.* 69.9%, log-rank *P* < 0.001; **Supplementary Fig. 1 A**). At 10 years after diagnosis, the cumulative incidence of cardiovascular death was 1.1% in HFimpEF patients and 11.1% in non-HFimpEF patients (**Supplementary Fig. 1B and 1C**). The time-dependent Cox regression analysis revealed that, after adjustment for sex, age, and HF etiology, the instantaneous risk of the primary outcome in patients experiencing LVEF improvement was 59% lower than observed while remaining in the HFrEF state (adjusted hazard ratio, aHR 0.41 [95% confidence interval, CI 0.27–0.63], *P* < 0.001; **Supplementary Table 2**).

### 3.3 Primary outcome

**Fig. 1:**
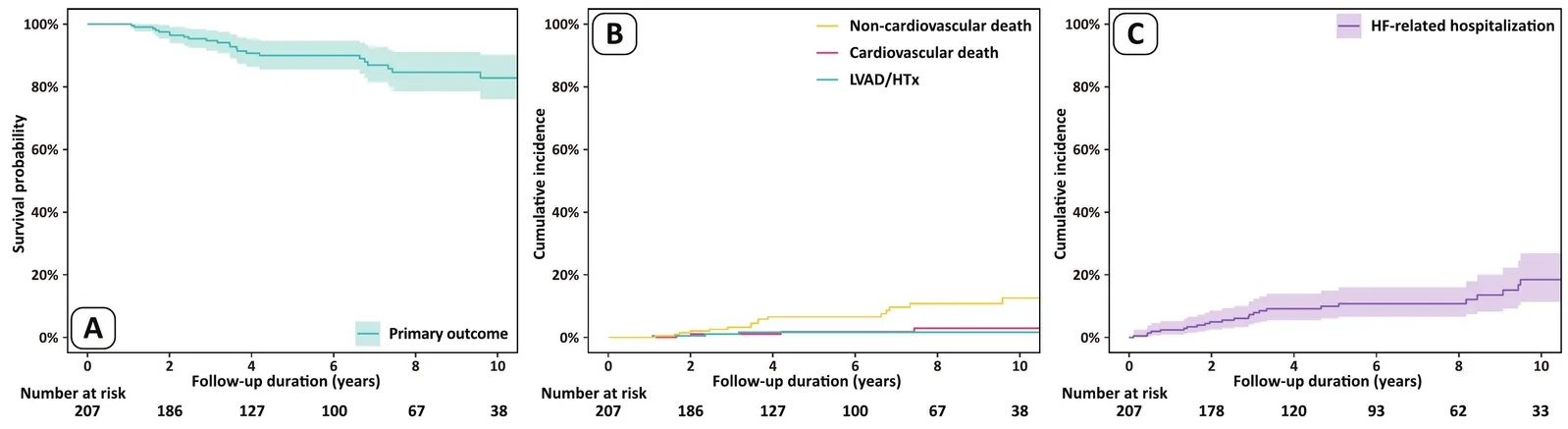
Survival analyses in heart failure patients with improved ejection fraction. A) The Kaplan-Meier curve demonstrated a primary outcome-free survival probability of 90.0% at 5 years and 82.8% at 10 years in the HFimpEF cohort. Primary outcome was defined as the composite of all-cause death, LVAD implantation, or heart transplantation. B) Competing-risk analysis showed that the cumulative incidence of cardiovascular death was 1.8% at 5 years and 2.9% at 10 years, while that of non-cardiovascular death was 6.6% at 5 years and 13.0% at 10 years. There were three cases undergoing LVAD implantation or heart transplantation during follow-up. C) The cumulative incidence of HF-related hospitalization was 10.0% at 5 years and 18.0% at 10 years. Follow-up duration started at the time of LVEF improvement. HF, heart failure; HFimpEF, heart failure with improved ejection fraction; HTx, heart transplantation; LVAD, left ventricular assist device; LVEF, left ventricular ejection fraction.

Table 2 summarizes the outcomes of HFimpEF patients after LVEF improvement. The duration from the time of LVEF improvement to the latest follow-up was 5.3 years (IQR 3.0,9.2). Twenty-five patients experienced the primary outcome, including 22 deaths, two cases of LVAD implantation, and one case of heart transplantation. The Kaplan-Meier curve demonstrated a primary outcome-free survival probability of 90.0% at 5 years and 82.8% at 10 years in the HFimpEF cohort after LVEF improvement (Fig. 1A). Competing-risk analysis showed that the post-improvement cumulative incidence of cardiovascular death was 1.8% at 5 years and 2.9% at 10 years, while that of non-cardiovascular death was 6.6% at 5 years and 13.0% at 10 years (Fig. 1B). Detailed information and causes of death for deceased patients are presented in Table 3. The median age of deceased patients was 64 years (IQR 52,71) at the time of LVEF improvement and 69 years (IQR 61,76) at the time of death, respectively. The median follow-up duration of these patients was 3.7 years (IQR 2.1,6.8). Non-cardiovascular causes accounted for 82% of deaths, with eleven patients dying from cancer and seven from other causes.

**Table 2:** Outcomes of heart failure patients with improved ejection fraction.

	Total population (*N* = 207)
**Follow-up duration, y**^a^	5.3 (3.0, 9.2)
**Primary outcome**	25 (12)
Death	22 (11)
Cardiovascular	4 (2)
Cancer	11 (5)
Other	7 (3)
LVAD implantation	2 (1)
Heart transplantation	1 (<1)
**HF-related hospitalization**	24 (12)
**Myocardial infarction**	8 (4)
**Atrial fibrillation**	23 (11)
**Life-threatening ventricular arrhythmias**	24 (12)
Anti-tachycardia pacing	10 (5)
Appropriate ICD shock	11 (5)
Ventricular tachycardia or fibrillation	3 (1)
**Cerebrovascular events**	9 (4)
Cerebrovascular accident	2 (1)
Transient ischemic attack	7 (3)
**CIED implantation**	9 (4)
Pacemakers	0
ICD	5 (2)
CRT-D	4 (2)

Data are median (interquartile range) or frequency (percentage). Percentages in parentheses represent crude event proportions and differ from the cumulative incidence estimated from survival analyses.

CIED, cardiac implantable electronic devices; CRT—D, cardiac resynchronization therapy with defibrillators; HF, heart failure; ICD, implantable cardioverter defibrillators; LVAD, left ventricular assist device; LVEF, left ventricular ejection fraction.

a Follow-up duration started at the time of LVEF improvement.

**Table 3:** Case information and causes of death for deceased patients with improved ejection fraction.

Patient number	Sex	Age at the time of LVEF improvement, y	Etiology	Drug treatment status	Follow-up duration, y^a^	Age at death, y	Cause of death	Contributing factors to death
1	Female	59	Toxic	RASi + BB + LD	1.6	61	Cardiovascular^b^	Decompensated heart failure
2	Male	62	Dilated	RASi + BB + MRA + LD	2.0	64	Cardiovascular	Decompensated heart failure
3	Female	69	Dilated	RASi + BB + LD + digoxin	4.2	73	Cardiovascular	Ventricular fibrillation
4	Male	70	Ischemic	RASi + BB + MRA + LD + digoxin	7.4	77	Cardiovascular	Decompensated heart failure
5	Female	39	Dilated	RASi + BB + MRA + LD	6.8	46	Cancer	Leukemia
6	Male	42	Dilated	RASi + BB + MRA	14.9	56	Cancer	Unknown malignancy
7	Male	45	Dilated	RASi + BB + MRA + LD + digoxin	2.0	47	Cancer	Lung cancer
8	Male	50	Toxic	RASi + BB + MRA + LD	2.9	53	Cancer	Lymphoma
9	Female	62	Toxic	RASi + BB + MRA + LD + digoxin	6.7	69	Cancer	Breast cancer
10	Female	63	Dilated	RASi + BB + MRA + LD + digoxin	3.7	67	Cancer^b^	Breast cancer
11	Female	66	Ischemic	RASi + BB + LD	3.8	70	Cancer^b^	Lung cancer
12	Female	71	Hypertensive	RASi + BB + LD + digoxin	3.4	74	Cancer	Unknown malignancy
13	Male	74	Ischemic	RASi + BB + LD	1.6	76	Cancer^b^	Prostate cancer
14	Male	78	Hypertensive	RASi + BB + LD + digoxin	1.1	79	Cancer	Rectal cancer
15	Female	79	Dilated	RASi + BB + LD + digoxin	1.7	81	Cancer^b^	Lymphoma
16	Male	41	Dilated	RASi + BB + MRA + LD	3.7	45	Other	Depression
17	Male	49	Dilated	RASi + BB + MRA + digoxin	12.0	61	Other	Alcoholism
18	Male	59	Dilated	RASi + BB	7.3	66	Other	Trauma
19	Female	70	Dilated	RASi + BB + MRA + LD	9.5	79	Other^b^	Unknown
20	Female	71	Hypertensive	RASi + BB	2.5	74	Other	Seizure
21	Female	72	Ischemic	RASi + BB + LD + digoxin	3.5	75	Other	Pneumonia
22	Male	75	Noncompaction	RASi + BB + MRA + LD + digoxin	6.7	81	Other	Unknown

BB, β-blockers; LD, loop diuretics; LVEF, left ventricular ejection fraction; MRA, mineralocorticoid receptor antagonists; RASi, renin-angiotensin system inhibitors.

a Follow-up duration started at the time of ejection fraction improvement.

b Patients with a prior diagnosis of cancer at the time of LVEF improvement.

### 3.4 Secondary outcomes

Following LVEF improvement, 24 patients experienced HF-related hospitalization (Table 2). When accounting for the primary outcome as a competing event, the cumulative incidence of HF-related hospitalization was 10.0% at 5 years and 18.0% at 10 years (Fig. 1C). New-onset myocardial infarction and atrial fibrillation occurred in 8 and 23 patients, respectively. A total of 24 patients experienced life-threatening ventricular arrhythmias. Cerebrovascular events were observed in nine patients, the majority of which were transient ischemic attacks. Nine patients underwent CIED implantation after LVEF improvement: six *de novo* implantations for primary prevention and three device upgrades.

### 3.5 Longitudinal trajectories

**Fig. 2:**
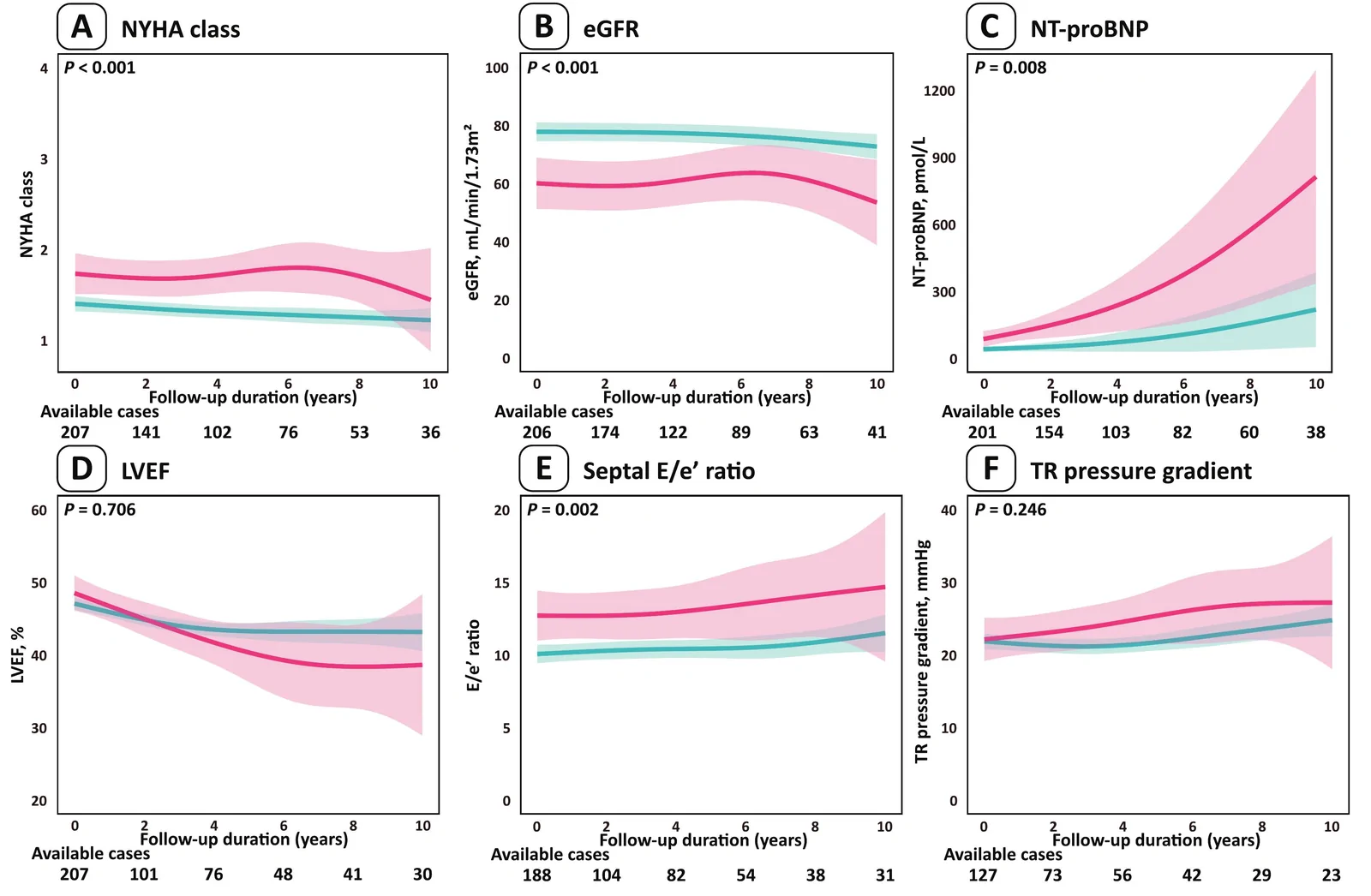
Longitudinal trajectories of key clinical parameters. The red curves depict the estimated trajectories in patients who experienced the primary outcome, whereas the green curves depict those in whom no primary outcome was observed during follow-up. The *P* value was obtained using the likelihood ratio test from the linear mixed-effects models, indicating overall between-group difference. Follow-up duration started at the time of LVEF improvement. eGFR, estimated glomerular filtration rate; LVEF, left ventricular ejection fraction; NT-proBNP, N-terminal pro-b-type natriuretic peptide; NYHA, New York Heart Association; TR, tricuspid regurgitation.

The longitudinal trajectories of selected clinical parameters are presented in Fig. 2; extended clinical parameters are shown in **Supplementary Fig. 2**. In general, HFimpEF patients who did not experience the primary outcome (*n* = 182, 88%) exhibited stable clinical performance following LVEF improvement. In contrast, the longitudinal trajectories fitted from the follow-up data of patients who experienced the primary outcome (*n* = 25, 12%) demonstrated a trend toward progressive clinical deterioration over time. At the time of LVEF improvement (follow-up year 0), while the LVEF was above 40% in both groups, differences were already apparent in several parameters, including NYHA class, estimated glomerular filtration rate (eGFR), and the septal E/e’ ratio (*P* < 0.050; Fig. 2A, B and E). Although NT-proBNP showed an increasing trend across the entire HFimpEF cohort, levels remained generally below approximately 300 pmol/L in the no-event group, whereas a relatively dramatic rise was observed in the group that experienced the primary outcome (*P* = 0.008; Fig. 2C). Over time, the estimated LVEF trajectory in patients with the primary outcome gradually declined to below 40%, whereas that in the no-event group remained stably above 40% throughout follow-up; however, the overall between-group difference was not statistically significant (*P* = 0.706; Fig. 2D). The trajectory of TR pressure gradient in both groups remained below 30 mmHg during follow-up (*P* = 0.246; Fig. 2F).

### 3.6 Regression analyses

After selection *via* univariable Cox regression and LASSO regression, six variables were included in the multivariable Cox regression analysis (**Supplementary Table 3**). Although age, history of chronic kidney disease, urea, and aspartate aminotransferase were associated with an increased risk of the primary outcome in the univariable Cox regression, these associations were not statistically significant after multivariable adjustment (*P* ≥ 0.050). In the multivariable regression, only history of cancer (aHR 2.72 [95% CI 1.11–6.65], *P* = 0.028) and septal E/e’ ratio (per 1 unit increase; aHR 1.09 [95% CI 1.03–1.16], *P* = 0.006) remained independent predictors of the primary outcome (Table 4).

**Table 4:** Cox regression analysis of primary outcome.

Variables (at the time of LVEF improvement)	Univariable Cox regression		Multivariable Cox regression	
	Unadjusted HR (95% CI)	*P* value	Adjusted HR (95% CI)	*P* value
Age (per 1 y)	1.06 (1.03–1.10)	**0.001**	1.02 (0.98–1.06)	0.307
Chronic kidney disease	4.22 (1.92–9.27)	**<0.001**	2.14 (0.71–6.39)	0.174
Cancer	3.56 (1.53–8.25)	**0.003**	2.72 (1.11–6.65)	**0.028**
Urea (per 1 mmol/L)	1.19 (1.09–1.28)	**<0.001**	1.08 (0.97–1.20)	0.163
AST (per 10 U/L)	1.30 (1.09–1.55)	**0.004**	1.22 (0.96–1.55)	0.104
E/e’ ratio (per 1 unit)	1.13 (1.05–1.21)	**0.001**	1.09 (1.03–1.16)	**0.006**

The variable selection for multivariable Cox regression analysis of primary outcome is presented in **Supplementary Table 3**.

AST, aspartate aminotransferase; CI, confidence interval; HR, hazard ratio; LVEF, left ventricular ejection fraction.

### 3.7 Subgroup analyses

During follow-up, 47 (23%) patients were dependent on RASi and β-blockers alone, whereas the remaining 160 (77%) patients were additionally prescribed at least one other drug. Patient characteristics at the time of LVEF improvement grouped by pharmacotherapy are presented in **Supplementary Table 4**. Overall, patients with RASi and β-blockers alone demonstrated a more favorable clinical profile at the time of LVEF improvement than those receiving additional HF pharmacotherapy, including younger age, fewer comorbidities, lower symptom burden (reflected by NYHA class), better renal function, lower NT-proBNP levels, shorter QRS duration, and slightly higher LVEF (all *P* < 0.050). Upon division of patients by pharmacotherapy, the RASi+β-blocker subgroup experienced fewer primary outcome events (2/47 *vs.* 23/160, log-rank *P* = 0.021) and HF-related rehospitalizations (1/47 *vs.* 23/160, *P* = 0.019) during a longer duration of follow-up (8.3 years [IQR 4.1,11.1] *vs.* 4.8 years [IQR 2.7,8.1], *P* < 0.001), compared to the RASi+β-blocker+others subgroup (**Supplementary Table 5**). Subgroup survival analysis demonstrated that the RASi+β-blocker subgroup had a higher 10-year event-free survival probability for death or advanced HF therapies (94.3% *vs.* 78.3%; **Supplementary Fig. 3**). The only two events observed in this subgroup were non-cardiovascular deaths, one due to trauma and another due to seizure (Table 3). Based on the Cox regression analysis, patients with NYHA class II/III/IV (aHR 0.45 [95%CI 0.20–0.91], *P* = 0.026) or larger LVEDD (per 1 mm increase; aHR 0.95 [95%CI 0.91–0.99], *P* = 0.017) were less likely to receive dual-drug therapy (**Supplementary Tables 6 and 7**).

When stratified HFimpEF patients by the magnitude of LVEF improvement, only 35 patients (17%) had an absolute improvement of <10% points. The 10-year primary outcome-free survival probability was comparable between patients with <10% and ≥ 10% improvement (86.4% *vs.* 82.3%, log-rank *P* = 0.951; **Supplementary Fig. 4 A**). The longitudinal LVEF trajectories also did not differ significantly (*P* = 0.077; **Supplementary Fig. 4B**).

## 4 Discussion

In this real-world observational HF cohort study, we described the clinical course of HFimpEF patients who reached a state of asymptomatic left ventricular dysfunction and evaluated their long-term outcome. We found that the long-term cardiovascular outcomes of HFimpEF patients were excellent and predominantly driven by non-cardiovascular death. Among patients who did not experience death or require advanced HF therapies (LVAD implantation/heart transplantation), multiple clinical parameters remained stable in the long term. In addition, patients treated with RASi and β-blockers alone appeared to maintain favorable outcomes during follow-up. Taken together, these findings provide important insights into the long-term clinical management of HFimpEF.

The novelty of the current study lies in its comprehensive characterization of HFimpEF over more than ten years of follow-up, not only delineating their clinical changes from initial HF diagnosis to LVEF improvement, but also characterizing the longitudinal trajectories of multiple clinical parameters thereafter. In our cohort, LVEF improvement was observed in 33% of HFrEF patients since HF diagnosis, falling within the previously reported prevalence range of 9% to 60% [7], [8], [9]. However, these estimates are largely influenced by the heterogeneity of HFimpEF definitions and study populations. Previous studies have suggested that patients with a non-ischemic etiology are more likely to experience left ventricular reverse remodeling [12], [21]. The vast majority (87%) in our HFimpEF cohort had a non-ischemic etiology, which is in line with this notion. A meta-analysis reported that HFimpEF is associated with an approximately 60% lower risk of mortality or hospitalization compared with HFrEF [22]. We further observed this significantly reduced risk associated with HFimpEF using a time-dependent Cox regression approach. The 10-year event-free survival probability for death or advanced HF therapies in our HFimpEF cohort was 82.8%. Such an encouraging result suggests that, with GRMT and long-term individualized management, HFimpEF patients may achieve an excellent prognosis [23].

Of note, the 10-year cumulative incidence of cardiovascular death was only 2.9% in our cohort, implying that the leading factor influencing survival in HFimpEF patients may not be cardiovascular events, but rather other underlying causes and comorbidities, such as aging and cancer [10], [12]. Our Cox regression corroborates this viewpoint by identifying the presence of cancer as an independent predictor of the primary outcome. Another independent predictor identified in our study was the E/e’ ratio, an important indicator of left ventricular filling pressure [24]. Its longitudinal trajectory further underscored its prognostic relevance: differences between patients who did and did not experience the primary outcome were already evident at the time of LVEF improvement, and this separation persisted throughout follow-up, with consistently lower values observed in those remaining free of events. The E/e’ ratio has been shown to aid in the assessment of congestion; in the setting of improved LVEF, it may provide particular predictive value for residual HF risk and major cardiovascular events [25], [26]. Although renal function parameters such as eGFR demonstrated a similarly discriminative pattern in the longitudinal trajectory, and a history of chronic kidney disease was also included as a covariate in the multivariable Cox regression, neither reached statistical significance. Further studies are therefore warranted to clarify the importance and predictive value of renal function in patients with HFimpEF.

A continuing controversy in HFimpEF is that echocardiography-derived LVEF is inherently dynamic, and some patients who initially meet the criteria for HFimpEF may subsequently deteriorate and be reclassified as HFrEF [2], [11], [12], [13]. This concept has been highlighted in prior studies and is also supported by our data. Specifically, the longitudinal LVEF trajectory in patients who died or required advanced HF therapies gradually declined to below 40%, suggesting that a subset of patients with HFimpEF remains vulnerable to relapse despite initial improvement. This indicates that defining or classifying “improvement” on the basis of LVEF alone is insufficient. Future risk stratification may need to integrate biomarkers (*e.g.*, E/e’ ratio and NT-proBNP) and CMR-derived parameters to better distinguish true myocardial recovery from temporary remission [3], [9]. In addition, greater attention should be paid to the role of reversible etiologies (*e.g.*, myocarditis, and chemotherapy-induced cardiomyopathy), as the durability of LVEF improvement likely depends on whether the underlying cause can be corrected [9].

In our subgroup analysis, the majority of patients (83%) had an absolute LVEF increase of ≥10% points at the time of improvement (median individual LVEF increase: 19% [IQR 13,26]). However, these patients did not demonstrate significantly better subsequent outcomes than those with <10% LVEF improvement. This suggests that achieving an LVEF >40%, rather than the magnitude of improvement alone, may be more relevant to subsequent prognosis, although this hypothesis requires validation in larger cohorts. Patients who were dependent on RASi and β-blockers alone during follow-up appeared to have an excellent prognosis, with only two non-cardiovascular deaths observed over a median follow-up of 8.3 years. Although exploratory and inherently limited by the observational nature of the study, with no intervention intentionally applied, these findings offer preliminary insights into the potential role of dual-drug regimen in HFimpEF [27]. While the TRED-HF trial [28] has demonstrated that complete withdrawal of multiple HF drugs is not feasible in this population, the optimal pharmacotherapeutic management of HFimpEF remains an area of clinical investigation [29]. The ongoing TRED-HF2 trial (NCT06091475) is investigating the feasibility of maintaining RASi and β-blockers alone in HFimpEF patients with dilated cardiomyopathy. As findings from future studies continue to emerge, we will eventually be able to determine how to mitigate polypharmacy-associated burden while preserving therapeutic efficacy.

### 4.1 Study limitations

Although the data were derived from a prospective real-world registry, the present study was conducted in a retrospective manner. To obtain granular follow-up data, the study population was restricted to a single tertiary referral HF center. Consequently, the registry may have included younger patients and a higher proportion of non-ischemic etiologies than the general HF population, potentially contributing to the comparatively favorable outcomes observed in this study [30]. The small sample size, declining number of available observations over time, and limited number of HF-related events constrained the number of variables that could be included in the regression models. In addition, the limited sample size and event rate precluded robust etiology-specific analyses, and we were therefore unable to reliably assess whether clinical trajectories and outcomes differed across specific underlying etiologies, including potentially reversible forms of cardiomyopathy. However, in the section of longitudinal trajectories, we applied linear mixed-effects models, which are well-suited for handling missing data while simultaneously capturing intra-correlations and nonlinearity in repeated measurements [17]. This methodological advantage advances the current literature by characterizing a broader set of clinical parameters and providing more robust trajectory estimates. Although our findings provide preliminary insights into the characteristics of HFimpEF patients with RASi+β-blocker dual-drug therapy (lower NYHA class and smaller LVEDD), the selection of patients in whom this treatment approach can be safely and effectively maintained requires validation in future prospective studies. Ultimately, because the Rijnmond HF registry was established prior to the emergence of SGLT-2i, the influence of this medication was not evaluated in our study. Nevertheless, analyses from the DELIVER trial suggest that SGLT-2i may be beneficial to HFimpEF patients [31]. Further investigations are warranted to reveal its impact on this population.

## 5 Conclusions

In this real-world registry, approximately one-third of HFrEF patients experienced LVEF improvement. The long-term outcome of HFimpEF patients was generally favorable, with most deaths attributable to non-cardiovascular causes. Among patients without death or need for advanced HF therapy, clinical parameters remained stable during follow-up. A history of cancer and an elevated E/e’ ratio independently predicted the composite of death and advanced HF therapy. A subgroup of patients with a more favorable clinical profile were treated with RASi and β-blockers alone following LVEF improvement; however, the efficacy and safety of this dual-drug regimen should be further validated in future trials before being recommended in routine long-term clinical practice.

## CRediT authorship contribution statement

**Yuxiang Luo:** Writing – original draft, Visualization, Methodology, Funding acquisition, Formal analysis, Data curation. **Yusuf Z. Sener:** Writing – review & editing, Validation. **Hongtao Tie:** Writing – review & editing, Validation, Methodology, Formal analysis. **Kevin M. Veen:** Writing – review & editing, Validation, Methodology, Formal analysis. **Mattie J. Lenzen:** Writing – review & editing, Project administration. **Alina A. Constantinescu:** Writing – review & editing, Investigation. **Olivier C. Manintveld:** Writing – review & editing, Investigation. **Robert M.A. van der Boon:** Writing – review & editing, Investigation. **Wouter C. Meijers:** Writing – review & editing, Investigation. **Dominic A. Theuns:** Writing – review & editing. **Osama Soliman:** Writing – review & editing. **Kadir Caliskan:** Writing – review & editing, Validation, Supervision, Resources, Methodology, Investigation, Funding acquisition, Conceptualization.

## Patient consent statement

The authors confirm that patient consent forms have been obtained for this article.

## Ethics statement

This study was performed in line with the principles of the Declaration of Helsinki. Approval was granted by the ethics committee of Erasmus University Medical Center (MEC-2014-100).

## Funding

This work was supported by grants from the 10.13039/501100004543China Scholarship Council [No. 202507720037 to Y.L.].

## Declaration of competing interest

The authors declare the following financial interests/personal relationships which may be considered as potential competing interests:

Yuxiang Luo reports financial support was provided by China Scholarship Council. If there are other authors, they declare that they have no known competing financial interests or personal relationships that could have appeared to influence the work reported in this paper.

## Data Availability

Data supporting the findings of this study are available from the corresponding author upon reasonable request.

## References

[1] Heidenreich P., Sandhu A. (2024). Advances in management of heart failure. BMJ.

[2] Pensa A.V., Khan S.S., Shah R.V., Wilcox J.E. (2024). Heart failure with improved ejection fraction: beyond diagnosis to trajectory analysis. Prog. Cardiovasc. Dis..

[3] Riccardi M., Pabon M.A., Bhatt A.S. (2025). Heart failure with improved ejection fraction: definitions, epidemiology, and management. J. Am. Coll. Cardiol..

[4] Heidenreich P.A., Bozkurt B., Aguilar D. (2022). 2022 AHA/ACC/HFSA guideline for the management of heart failure: a report of the American College of Cardiology/American Heart Association joint committee on clinical practice guidelines. Circulation.

[5] McDonagh T.A., Metra M., Adamo M. (2022). 2021 ESC guidelines for the diagnosis and treatment of acute and chronic heart failure: developed by the task force for the diagnosis and treatment of acute and chronic heart failure of the European Society of Cardiology (ESC). With the special contribution of the Heart Failure Association (HFA) of the ESC. Eur. J. Heart Fail..

[6] Walsh M.N., Kober L., Sliwa K. (2026). AHA/ACC/ESC/WHF expert consensus document: second universal definition of heart failure (2026). Circulation.

[7] Veltmann C., Duncker D., Doering M. (2024). Therapy duration and improvement of ventricular function in de novo heart failure: the Heart Failure Optimization study. Eur. Heart J..

[8] Florea V.G., Rector T.S., Anand I.S., Cohn J.N. (2016). Heart failure with improved ejection fraction: clinical characteristics, correlates of recovery, and survival: results from the valsartan heart failure trial. Circ. Heart Fail..

[9] Kodur N., Tang W.H.W. (2025). Management of heart failure with improved ejection fraction: current evidence and controversies. JACC Heart Fail..

[10] de Groote P., Fertin M., Duva Pentiah A., Goéminne C., Lamblin N., Bauters C. (2014). Long-term functional and clinical follow-up of patients with heart failure with recovered left ventricular ejection fraction after β-blocker therapy. Circ. Heart Fail..

[11] Hammer Y., Yosef M., Khalatbari S., Aaronson K.D. (2023). Heart failure with recovered ejection fraction in patients with nonischemic cardiomyopathy: characteristics, outcomes, and long-term follow-up. J. Card. Fail..

[12] Manca P., Stolfo D., Merlo M. (2022). Transient versus persistent improved ejection fraction in non-ischaemic dilated cardiomyopathy. Eur. J. Heart Fail..

[13] Takigahira T., Nochioka K., Miyata S. (2026). Prognostic significance of subsequent decline in LVEF in heart failure with improved ejection fraction - a report from the CHART-2 study. Int. J. Cardiol. Heart. Vasc..

[14] Hulot J.S., Ter Maaten J.M., Bayes-Genis A. (2025). Heart failure improvement, remission, and recovery: a European Journal of Heart Failure expert consensus document. Eur. J. Heart Fail..

[15] Ponikowski P., Voors A.A., Anker S.D. (2016). 2016 ESC guidelines for the diagnosis and treatment of acute and chronic heart failure: the Task Force for the diagnosis and treatment of acute and chronic heart failure of the European Society of Cardiology (ESC) Developed with the special contribution of the Heart Failure Association (HFA) of the ESC. Eur. Heart J..

[16] Hicks K.A., Mahaffey K.W., Mehran R. (2018). 2017 cardiovascular and stroke endpoint definitions for clinical trials. J. Am. Coll. Cardiol..

[17] Wang X., Andrinopoulou E.R., Veen K.M., Bogers A., Takkenberg J.J.M. (2022). Statistical primer: an introduction to the application of linear mixed-effects models in cardiothoracic surgery outcomes research-a case study using homograft pulmonary valve replacement data. Eur. J. Cardiothorac. Surg..

[18] Cox D.R. (1972). Regression models and life-tables. J. R. Stat. Soc. B. Methodol..

[19] Tibshirani R. (1996). Regression shrinkage and selection via the lasso. J. R. Stat. Soc. Ser. B Stat Methodol..

[20] White I.R., Royston P., Wood A.M. (2011). Multiple imputation using chained equations: issues and guidance for practice. Stat. Med..

[21] Lupón J., Díez-López C., de Antonio M. (2017). Recovered heart failure with reduced ejection fraction and outcomes: a prospective study. Eur. J. Heart Fail..

[22] He Y., Ling Y., Guo W. (2021). Prevalence and prognosis of HFimpEF developed from patients with heart failure with reduced ejection fraction: systematic review and meta-analysis. Front. Cardiovasc. Med..

[23] Min K.H., Go A.S., Parikh R.V. (2025). Clinical characteristics, early GDMT initiation, and likelihood of heart failure with improved ejection fraction. J. Card. Fail..

[24] Nagueh S.F., Smiseth O.A., Appleton C.P. (2016). Recommendations for the evaluation of left ventricular diastolic function by echocardiography: an update from the American Society of Echocardiography and the European Association of Cardiovascular Imaging. J. Am. Soc. Echocardiogr..

[25] Anastasiou V., Peteinidou E., Moysidis D.V. (2024). Multiorgan congestion assessment by venous excess ultrasound score in acute heart failure. J. Am. Soc. Echocardiogr..

[26] Wu V.C., Huang Y.C., Wang C.L. (2023). Association of echocardiographic parameter E/e’ with cardiovascular events in a diverse population of inpatients and outpatients with and without cardiac diseases and risk factors. J. Am. Soc. Echocardiogr..

[27] Luo Y., Ghossein M.A., Caliskan K. (2025). Pharmacotherapy for patients with heart failure and improved ejection fraction: updating our understanding. J. Am. Coll. Cardiol..

[28] Halliday B.P., Wassall R., Lota A.S. (2019). Withdrawal of pharmacological treatment for heart failure in patients with recovered dilated cardiomyopathy (TRED-HF): an open-label, pilot, randomised trial. Lancet.

[29] Luo Y., Xiao W., Sener Y.Z. (2025). Minimization or withdrawal of oral pharmacotherapy in chronic heart failure patients with improved myocardial function: a systematic review. Eur. J. Heart Fail..

[30] Linssen G.C.M., Veenis J.F., Brunner-La Rocca H.P. (2020). Differences in guideline-recommended heart failure medication between Dutch heart failure clinics: an analysis of the CHECK-HF registry. Neth. Hear. J..

[31] Vardeny O., Fang J.C., Desai A.S. (2022). Dapagliflozin in heart failure with improved ejection fraction: a prespecified analysis of the DELIVER trial. Nat. Med..

