# Supplementary material for: Long-term outcome of heart failure patients with improved ejection fraction: Results from a real-world registry
Source: Int J Cardiol Heart Vasc. 2026 Sep 15;66:102008. doi: 10.1016/j.ijcha.2026.102008 (PMC13598516; doi:10.1016/j.ijcha.2026.102008)
Supplement: Supplementary file 1 [file mmc1.docx]

**Long-term outcome of heart failure patients with improved ejection fraction: results from a real-world registry**

**Supplementary Materials**

**Supplementary Figure 1** Survival analyses by heart failure phenotype

**Supplementary Figure 2** Longitudinal trajectories of extended clinical parameters

**Supplementary Figure 3** Subgroup survival analysis of primary outcome by pharmacotherapy

**Supplementary Figure 4** Subgroup analyses by the magnitude of LVEF improvement

**Supplementary Table 1** Patient characteristics and outcomes by heart failure phenotype

**Supplementary Table 2** Time-dependent Cox regression analysis of primary outcome

**Supplementary Table 3** Variable selection for Cox regression analysis of primary outcome

**Supplementary Table 4** Subgroup patient characteristics at LVEF improvement by pharmacotherapy

**Supplementary Table 5** Subgroup outcomes by pharmacotherapy

**Supplementary Table 6** Variable selection for Cox regression analysis of dual-drug therapy

**Supplementary Table 7** Cox regression analyses of dual-drug therapy

**Supplementary figure captions**

**Supplementary Figure 1** Survival analyses by heart failure phenotype. A) The 10-year primary outcome-free survival probability from the time of heart failure diagnosis was 91.9% in HFimpEF patients and 69.9% in non-HFimpEF patients (Log-rank P<0.001). Primary outcome was defined as the composite of all-cause death, LVAD implantation, or heart transplantation. B) Competing-risk analysis showed that the cumulative incidence of cardiovascular death in HFimpEF patients was 1.1% at 10 years after heart failure diagnosis. C) The cumulative incidence of cardiovascular death in non-HFimpEF patients was 11.1% at 10 years after heart failure diagnosis. Time zero was the date of heart failure diagnosis.

HF, heart failure; HFimpEF, heart failure with improved ejection fraction; HTx, heart transplantation; LVAD, left ventricular assist device.

**Supplementary Figure 2** Longitudinal trajectories of extended clinical parameters. The red curves depict the estimated trajectories in patients who experienced the primary outcome, whereas the green curves depict those in whom no primary outcome was observed during follow-up. RV function was visually assessed by echocardiography and graded as normal function, mild, moderate, or severe dysfunction, coded as 1, 2, 3, and 4, respectively. The *P* value was obtained using the likelihood ratio test from the linear mixed-effects models, indicating overall between-group difference. Follow-up duration started at the time of LVEF improvement.

LA, left atrium; LVEDD, left ventricular end-diastolic diameter; LVEF, left ventricular ejection fraction; LVESD, left ventricular end-systolic diameter; RV, right ventricle; TAPSE, tricuspid annular plane systolic excursion; VCI, vena cava inferior.

**Supplementary Figure 3** Subgroup survival analysis of primary outcome by pharmacotherapy. The RASi+β-blocker subgroup comprised patients who were dependent on RASi and β-blockers alone during follow-up (dual-drug therapy). The RASi+β-blocker+others subgroup comprised patients who remained on RASi and β-blockers but were additionally prescribed at least one other drug during follow-up, including mineralocorticoid receptor antagonists, loop diuretics, and digoxin. The 10-year primary outcome-free survival probability was 94.3% in the RASi+β-blocker subgroup and 78.3% in the RASi+β-blocker+others subgroup (log-rank *P*=0.021). Follow-up duration started at the time of LVEF improvement.

LVEF, left ventricular ejection fraction; RASi, renin-angiotensin system inhibitors.

**Supplementary Figure 4** Subgroup analyses by the magnitude of LVEF improvement. A) The 10-year primary outcome-free survival probability was 86.4% in the <10% LVEF improvement subgroup and 82.3% in the ≥10% LVEF improvement subgroup (log-rank *P*=0.951). B) Longitudinal LVEF trajectories in HFimpEF patients stratified by the magnitude of LVEF improvement. The *P* value was obtained using the likelihood ratio test from the linear mixed-effects models, indicating overall between-group difference. Follow-up duration started at the time of LVEF improvement.

HFimpEF, heart failure with improved ejection fraction; LVEF, left ventricular ejection fraction.

**Supplementary Figure 1** Survival analyses by heart failure phenotype

**Supplementary Figure 2** Longitudinal trajectories of extended clinical parameters

**Supplementary Figure 3** Subgroup survival analysis of primary outcome by pharmacotherapy

**Supplementary Figure 4** Subgroup analyses by the magnitude of LVEF improvement

**Supplementary Table 1** Patient characteristics and outcomes by heart failure phenotype

|  | **HFimpEF**  **(N=207)** | **Non-HFimpEF**  **(N=428)** | ***P* value** |
| --- | --- | --- | --- |
| **Patient characteristics** |  |  |  |
| **Male** | 118 (57) | 307 (72) | **<0.001** |
| **Age at heart failure diagnosis, y** | 49 (37, 59) | 51 (41, 59) | 0.214 |
| **Ischemic etiology** | 26 (13) | 170 (40) | **<0.001** |
| **Outcomes** |  |  |  |
| **Follow-up duration since heart failure diagnosis, y** | 10.4 (6.2, 14.9) | 10.3 (5.6, 14.8) | 0.543 |
| **Primary outcome** | 25 (12) | 234 (55) | **<0.001** |
| Death | 22 (11) | 132 (31) |  |
| Cardiovascular | 4 (2) | 95 (22) |  |
| Non-cardiovascular | 18 (9) | 37 (9) |  |
| LVAD implantation/Heart transplantation | 3 (1) | 102 (24) |  |

Data are median (interquartile range) or frequency (percentage). Percentages in parentheses represent crude event proportions and differ from the cumulative incidence estimated from survival analyses.

HFimpEF, heart failure with improved ejection fraction; LVAD, left ventricular assist device.

**Supplementary Table 2** Time-dependent Cox regression analysis of primary outcome

| **Variables** | **Multivariable Cox regression** | |
| --- | --- | --- |
|  | **Adjusted HR (95% CI)** | ***P* value** |
| **Sex** |  |  |
| Female | Reference |  |
| Male | 1.21 (0.91 - 1.59) | 0.184 |
| **Age at heart failure diagnosis (per 1 y)** | 1.04 (1.03 - 1.05) | **<0.001** |
| **Etiology** |  |  |
| NICM | Reference |  |
| ICM | 0.94 (0.72 - 1.23) | 0.669 |
| **Time-dependent heart failure state** |  |  |
| HFrEF | Reference |  |
| HFimpEF | 0.41 (0.27 - 0.63) | **<0.001** |

Heart failure state was modeled as a time-dependent covariate. Time zero was the date of heart failure diagnosis. Patients who subsequently developed HFimpEF contributed person-time to the HFrEF state before the date of ejection fraction improvement and to the HFimpEF state thereafter; patients without improvement contributed all person-time to the HFrEF state.

CI, confidence interval; HFimpEF, heart failure with improved ejection fraction; HFrEF, heart failure with reduced ejection fraction; HR, hazard ratio; ICM, ischemic cardiomyopathy; NICM, non-ischemic cardiomyopathy.

**Supplementary Table 3** Variable selection for Cox regression analysis of primary outcome

| **Variables (at the time of LVEF improvement)** | **Univariable Cox regression** | | **LASSO regression** | **Multivariable Cox regression** | |
| --- | --- | --- | --- | --- | --- |
|  | **Unadjusted HR (95% CI)** | ***P* value** | **Coefficients (β)** | **Adjusted HR (95% CI)** | ***P* value** |
| **Demographics** |  |  |  |  |  |
| **Sex** |  |  |  |  |  |
| Female | Reference |  |  |  |  |
| Male | 0.72 (0.33 - 1.58) | 0.406 | — |  |  |
| **Age (per 1 y)** | 1.06 (1.03 - 1.10) | **0.001** | **0.002** | 1.02 (0.98 - 1.06) | 0.307 |
| **Body mass index (per 1 kg/m²)** | 0.98 (0.90 - 1.07) | 0.634 | — |  |  |
| **Race** |  |  |  |  |  |
| Non-Caucasian | Reference |  |  |  |  |
| Caucasian | 0.71 (0.24 - 2.10) | 0.539 | — |  |  |
| **Etiology** |  |  |  |  |  |
| NICM | Reference |  |  |  |  |
| ICM | 1.13 (0.34 - 3.78) | 0.847 | — |  |  |
| **Medical history** |  |  |  |  |  |
| **Hypertension** | 2.09 (0.92 - 4.75) | 0.078 |  |  |  |
| **Atrial fibrillation** | 1.76 (0.73 - 4.24) | 0.208 |  |  |  |
| **Cerebrovascular events** | 0.91 (0.21 - 3.87) | 0.894 |  |  |  |
| **Diabetes mellitus** | 2.79 (0.94 - 8.29) | 0.066 |  |  |  |
| **Chronic obstructive pulmonary disease** | 1.99 (0.68 - 5.82) | 0.206 |  |  |  |
| **Obstructive sleep apnea syndrome** | 0.80 (0.11 - 5.95) | 0.829 |  |  |  |
| **Chronic kidney disease** | 4.22 (1.92 - 9.27) | **<0.001** | **0.052** | 2.14 (0.71 - 6.39) | 0.174 |
| **Cancer** | 3.56 (1.53 - 8.25) | **0.003** | **0.294** | 2.72 (1.11 - 6.65) | **0.028** |
| **CIED implantation** |  |  |  |  |  |
| None | Reference |  |  |  |  |
| Any CIED implantation | 0.69 (0.31 - 1.52) | 0.356 |  |  |  |
| **Clinical parameters** |  |  |  |  |  |
| **Duration from diagnosis to improvement (per 1 y)** | 1.07 (0.99 - 1.15) | 0.074 |  |  |  |
| **NYHA class** |  |  |  |  |  |
| NYHA class I | Reference |  |  |  |  |
| NYHA class II/III/IV | 2.67 (1.19 - 5.97) | **0.017** | — |  |  |
| **Heart rate (per 10 bpm)** | 1.26 (0.96 - 1.64) | 0.094 |  |  |  |
| **Systolic blood pressure (per 1 mmHg)** | 1.01 (0.99 - 1.03) | 0.266 |  |  |  |
| **Diastolic blood pressure (per 1 mmHg)** | 1.02 (0.97 - 1.06) | 0.484 |  |  |  |
| **QRS duration (per 1 ms)** | 1.01 (1.00 - 1.02) | 0.121 |  |  |  |
| **Medication use** |  |  |  |  |  |
| **MRA** | 1.62 (0.67 - 3.89) | 0.282 |  |  |  |
| **Loop diuretics** | 3.64 (1.09 - 12.16) | **0.036** | — |  |  |
| **Digoxin** | 2.85 (1.26 - 6.45) | **0.012** | — |  |  |
| **Laboratory parameters** |  |  |  |  |  |
| **Glucose (per 1 mmol/L)** | 1.09 (0.91 - 1.30) | 0.347 |  |  |  |
| **eGFR (per 10 mL/min/1.73m²)** | 0.71 (0.60 - 0.84) | **<0.001** | — |  |  |
| **Urea (per 1 mmol/L)** | 1.19 (1.09 - 1.28) | **<0.001** | **0.071** | 1.08 (0.97 - 1.20) | 0.163 |
| **Sodium (per 1 mmol/L)** | 0.89 (0.75 - 1.05) | 0.153 |  |  |  |
| **Potassium (per 1 mmol/L)** | 0.50 (0.19 - 1.30) | 0.154 |  |  |  |
| **AST (per 10 U/L)** | 1.30 (1.09 - 1.55) | **0.004** | **0.001** | 1.22 (0.96 - 1.55) | 0.104 |
| **ALT (per 10 U/L)** | 1.11 (0.90 - 1.38) | 0.336 |  |  |  |
| **NT-proBNP (per 10 pmol/L)** | 1.04 (1.01 - 1.07) | **0.004** | — |  |  |
| **Hemoglobin (per 1 mmol/L)** | 0.70 (0.49 - 1.00) | 0.050 |  |  |  |
| **Imaging parameters** |  |  |  |  |  |
| **LA diameter (per 1 mm)** | 1.06 (1.00 - 1.13) | **0.049** | — |  |  |
| **LVEDD (per 1 mm)** | 0.99 (0.94 - 1.05) | 0.730 |  |  |  |
| **LVEF (per 1 %)** | 1.03 (0.96 - 1.10) | 0.455 |  |  |  |
| **VCI diameter (per 1 mm)** | 0.97 (0.87 - 1.07) | 0.519 |  |  |  |
| **TAPSE (per 1 mm)** | 0.94 (0.87 - 1.03) | 0.172 |  |  |  |
| **Septal E/e’ ratio (per 1 unit)** | 1.13 (1.05 - 1.21) | **0.001** | **0.031** | 1.09 (1.03 - 1.16) | **0.006** |
| **RV dysfunction** | 2.21 (0.94 - 5.16) | 0.068 |  |  |  |

Demographic variables and other statistically significant variables in the univariable Cox regression analysis underwent LASSO regression. Only variables with non-zero coefficients were included in the multivariable Cox regression analysis. The dash symbol indicates variables excluded by LASSO regression due to coefficient shrinkage to zero.

ALT, alanine aminotransferase; AST, aspartate aminotransferase; CI, confidence interval; CIED, cardiac implantable electronic devices; eGFR, estimated glomerular filtration rate; HR, hazard ratio; ICM, ischemic cardiomyopathy; LA, left atrium; LASSO, least absolute shrinkage and selection operator; LVEDD, left ventricular end-diastolic diameter; LVEF, left ventricular ejection fraction; LVESD, left ventricular end-systolic diameter; MRA, mineralocorticoid receptor antagonists; NICM, non-ischemic cardiomyopathy; NT-proBNP, N-terminal pro-b-type natriuretic peptide; NYHA, New York Heart Association; RV, right ventricle; TAPSE, tricuspid annular plane systolic excursion; VCI, vena cava inferior.

**Supplementary Table 4** Subgroup patient characteristics at LVEF improvement by pharmacotherapy

|  | **RASi+β-blocker**  **(n=47)** | **RASi+β-blocker+others**  **(n=160)** | ***P* value** |
| --- | --- | --- | --- |
| **Demographics** |  |  |  |
| **Male** | 25 (53) | 93 (58) | 0.616 |
| **Age, y** | 45 (38, 58) | 55 (46, 65) | **<0.001** |
| **Body mass index, kg/m²** | 26.2 (24.5, 30.3) | 26.1 (23.4, 29.8) | 0.512 |
| **Race** |  |  | 0.096 |
| Caucasian | 39 (83) | 145 (91) |  |
| African | 4 (9) | 5 (3) |  |
| Asian | 3 (6) | 3 (2) |  |
| Other | 1 (2) | 7 (4) |  |
| **Etiology** |  |  | **0.002** |
| Ischemic | 2 (4) | 24 (15) |  |
| Dilated | 19 (40) | 86 (54) |  |
| Hypertensive | 5 (11) | 4 (3) |  |
| Noncompaction | 13 (28) | 13 (8) |  |
| Myocarditis | 3 (6) | 6 (4) |  |
| Toxic | 1 (2) | 12 (8) |  |
| Peripartum | 0 | 3 (2) |  |
| Other | 4 (9) | 12 (8) |  |
| **Medical history** |  |  |  |
| **Hypertension** | 9 (19) | 43 (27) | 0.341 |
| **Atrial fibrillation** | 5 (11) | 39 (24) | **0.044** |
| **Cerebrovascular events** |  |  | 0.422 |
| Cerebrovascular accident | 1 (2) | 12 (8) |  |
| Transient ischemic attack | 2 (4) | 9 (6) |  |
| **Diabetes mellitus** | 0 | 21 (13) | **0.005** |
| **Chronic obstructive pulmonary disease** | 1 (2) | 15 (9) | 0.127 |
| **Obstructive sleep apnea syndrome** | 0 | 11 (7) | 0.073 |
| **Chronic kidney disease** | 5 (11) | 60 (38) | **<0.001** |
| **Cancer** | 3 (6) | 22 (14) | 0.211 |
| **CIED implantation** |  |  | **0.049** |
| Pacemakers | 1 (2) | 3 (2) |  |
| ICD | 14 (30) | 55 (34) |  |
| CRT-D | 4 (9) | 37 (23) |  |
| **Clinical parameters** |  |  |  |
| **Duration from diagnosis to improvement, y** | 1.7 (1.1, 4.7) | 3.6 (1.2, 7.3) | **0.036** |
| **NYHA class** |  |  | **0.003** |
| I | 38 (81) | 89 (56) |  |
| II | 9 (19) | 60 (38) |  |
| III | 0 | 11 (7) |  |
| **Heart rate, bpm** | 64 (59, 70) | 66 (60, 78) | 0.085 |
| **Systolic blood pressure, mmHg** | 120 (110, 130) | 120 (110, 137) | 0.874 |
| **Diastolic blood pressure, mmHg** | 75 (70, 80) | 71 (70, 80) | 0.332 |
| **QRS duration, ms** | 101 (93, 118) | 113 (101, 146) | **0.003** |
| **Medication use** |  |  |  |
| **RASi** |  |  | 0.477 |
| ACEi | 35 (75) | 104 (65) |  |
| ARB | 7 (15) | 28 (18) |  |
| ARNi | 5 (11) | 28 (18) |  |
| **MRA** | 22 (47) | 116 (73) | **0.001** |
| **Loop diuretics** | 27 (57) | 121 (76) | **0.026** |
| **Digoxin** | 10 (21) | 71 (44) | **0.004** |
| **Laboratory parameters** |  |  |  |
| **Glucose, mmol/L (n=177)** | 5.3 (4.9, 6.4) | 5.8 (5.2, 6.7) | 0.058 |
| **eGFR, mL/min/1.73m² (n=206)** | 80 (67, 93) | 70 (53, 89) | **0.008** |
| **Urea, mmol/L (n=205)** | 5.8 (4.6, 7.2) | 6.9 (5.1, 8.9) | **0.010** |
| **Sodium, mmol/L (n=206)** | 141 (140, 143) | 141 (139, 142) | 0.334 |
| **Potassium, mmol/L (n=206)** | 4.4 (4.2, 4.7) | 4.4 (4.1, 4.6) | 0.452 |
| **Total bilirubin, μmol/L (n=157)** | 8 (7, 10) | 8 (6, 11) | 0.599 |
| **AST, U/L (n=189)** | 24 (22, 29) | 25 (20, 32) | 0.657 |
| **ALT, U/L (n=190)** | 24 (17, 35) | 25 (19, 35) | 0.618 |
| **NT-proBNP, pmol/L (n=201)** | 12 (8, 36) | 26 (13, 57) | **0.001** |
| **Hemoglobin, mmol/L (n=191)** | 8.7 (8.0, 9.2) | 8.6 (7.9, 9.2) | 0.768 |
| **Imaging parameters** |  |  |  |
| **LA diameter, mm (n=195)** | 38 (35, 42) | 40 (36, 45) | 0.054 |
| **LVEDD, mm (n=193)** | 54 (49, 58) | 55 (51, 61) | 0.061 |
| **LVESD, mm (n=93)** | 36 (34, 43) | 42 (38, 46) | **0.006** |
| **LVEF, %** | 48 (45, 51) | 45 (43, 50) | **0.016** |
| **VCI diameter, mm (n=168)** | 17 (14, 18) | 16 (14, 19) | 0.473 |
| **TAPSE, mm (n=167)** | 23 (20, 26) | 22 (18, 25) | 0.191 |
| **Septal E/e’, ratio (n=188)** | 9.1 (7.7, 10.9) | 9.3 (7.3, 12.0) | 0.789 |
| **TR pressure gradient, mmHg (n=127)** | 19 (18, 23) | 23 (19, 25) | **0.016** |
| **RV dysfunction (n=166)** | 6/32 (19) | 31/134 (23) | 0.813 |
| **LGE pattern in CMR (n=67)** |  |  | 0.449 |
| None | 12 (71) | 26 (52) |  |
| Subendocardial | 1 (6) | 2 (4) |  |
| Midmyocardial | 3 (18) | 18 (36) |  |
| Transmural | 1 (6) | 4 (8) |  |

Data are median (interquartile range) or frequency (percentage). Available cases of variables with missing values are displayed in the row names. The RASi+β-blocker subgroup comprised patients who were dependent on RASi and β-blockers alone during follow-up (dual-drug therapy). The RASi+β-blocker+others subgroup comprised patients who remained on RASi and β-blockers but were additionally prescribed at least one other drug during follow-up, including MRA, loop diuretics, and digoxin.

ACEi, angiotensin-converting enzyme inhibitors; ALT, alanine aminotransferase; ARB, angiotensin II receptor blockers; ARNi, angiotensin receptor-neprilysin inhibitors; AST, aspartate aminotransferase; CIED, cardiac implantable electronic devices; CMR, cardiac magnetic resonance; CRT-D, cardiac resynchronization therapy with defibrillators; eGFR, estimated glomerular filtration rate; ICD, implantable cardioverter defibrillators; LA, left atrium; LGE, late gadolinium enhancement; LVEDD, left ventricular end-diastolic diameter; LVEF, left ventricular ejection fraction; LVESD, left ventricular end-systolic diameter; MRA, mineralocorticoid receptor antagonists; NT-proBNP, N-terminal pro-b-type natriuretic peptide; NYHA, New York Heart Association; RASi, renin-angiotensin system inhibitors; RV, right ventricle; TAPSE, tricuspid annular plane systolic excursion; TR, tricuspid regurgitation; VCI, vena cava inferior.

**Supplementary Table 5** Subgroup outcomes by pharmacotherapy

|  | **RASi+β-blocker**  **(n=47)** | **RASi+β-blocker+others**  **(n=160)** | ***P* value** |
| --- | --- | --- | --- |
| **Follow-up duration, y**^a^ | 8.3 (4.1, 11.1) | 4.8 (2.7, 8.1) | **<0.001** |
| **Primary outcome** | 2 (4) | 23 (14) | **0.021**^b^ |
| Death | 2 (4) | 20 (13) | 0.117 |
| Cardiovascular | 0 | 4 (3) |  |
| Cancer | 0 | 11 (7) |  |
| Other | 2 (4) | 5 (3) |  |
| LVAD implantation | 0 | 2 (1) |  |
| Heart transplantation | 0 | 1 (<1) |  |
| **HF-related hospitalization** | 1 (2) | 23 (14) | **0.019** |
| **Myocardial infarction** | 1 (2) | 7 (4) | 0.686 |
| **Atrial fibrillation** | 2 (4) | 21 (13) | 0.114 |
| **Life-threatening ventricular arrhythmias** | 4 (9) | 20 (13) | 0.607 |
| Anti-tachycardia pacing | 2 (4) | 8 (5) |  |
| Appropriate ICD shock | 2 (4) | 9 (6) |  |
| Ventricular tachycardia or fibrillation | 0 | 3 (2) |  |
| **Cerebrovascular events** | 2 (4) | 7 (4) | 1.000 |
| Cerebrovascular accident | 0 | 2 (1) |  |
| Transient ischemic attack | 2 (4) | 5 (3) |  |
| **CIED implantation** | 1 (2) | 8 (5) | 0.682 |
| Pacemakers | 0 | 0 |  |
| ICD | 0 | 5 (3) |  |
| CRT-D | 1 (2) | 3 (2) |  |

Data are median (interquartile range) or frequency (percentage). Percentages in parentheses represent crude event proportions and differ from the cumulative incidence estimated from survival analyses. The RASi+β-blocker subgroup comprised patients who were dependent on RASi and β-blockers alone during follow-up (dual-drug therapy). The RASi+β-blocker+others subgroup comprised patients who remained on RASi and β-blockers but were additionally prescribed at least one other drug during follow-up, including MRA, loop diuretics, and digoxin.

^a^ Follow-up duration started at the time of LVEF improvement.

^b^ *P* value of the primary outcome was derived from the log-rank test.

CIED, cardiac implantable electronic devices; CRT-D, cardiac resynchronization therapy with defibrillators; HF, heart failure; ICD, implantable cardioverter defibrillators; LVAD, left ventricular assist device; RASi, renin-angiotensin system inhibitors.

**Supplementary Table 6** Variable selection for Cox regression analysis of dual-drug therapy

| **Variables (at the time of LVEF improvement)** | **Univariable Cox regression** | | **LASSO regression** | **Multivariable Cox regression** | |
| --- | --- | --- | --- | --- | --- |
|  | **Unadjusted HR (95% CI)** | ***P* value** | **Coefficients (β)** | **Adjusted HR (95% CI)** | ***P* value** |
| **Demographics** |  |  |  |  |  |
| **Sex** |  |  |  |  |  |
| Female | Reference |  |  |  |  |
| Male | 0.89 (0.50 - 1.57) | 0.678 | — |  |  |
| **Age (per 1 y)** | 0.97 (0.95 - 0.99) | **0.005** | **-0.005** | 0.99 (0.97 - 1.01) | 0.353 |
| **Body mass index (per 1 kg/m²)** | 1.03 (0.97 - 1.09) | 0.396 | — |  |  |
| **Race** |  |  |  |  |  |
| Non-Caucasian | Reference |  |  |  |  |
| Caucasian | 0.60 (0.28 - 1.29) | 0.192 | — |  |  |
| **Etiology** |  |  |  |  |  |
| NICM | Reference |  |  |  |  |
| ICM | 0.31 (0.07 - 1.27) | 0.104 | — |  |  |
| **Medical history** |  |  |  |  |  |
| **Hypertension** | 0.83 (0.40 - 1.73) | 0.623 |  |  |  |
| **Atrial fibrillation** | 0.42 (0.17 - 1.06) | 0.065 |  |  |  |
| **Cerebrovascular events** | 0.85 (0.31 - 2.39) | 0.764 |  |  |  |
| **Diabetes mellitus** | 0.10 (0.01 - 0.70) | **0.013** | **-0.002** | 0.17 (0.00 - 1.27) | 0.099 |
| **Chronic obstructive pulmonary disease** | 0.25 (0.03 - 1.81) | 0.171 |  |  |  |
| **Obstructive sleep apnea syndrome** | 0.23 (0.01 - 1.57) | 0.170 |  |  |  |
| **Chronic kidney disease** | 0.20 (0.06 - 0.63) | **0.006** | **-0.307** | 0.40 (0.10 - 1.10) | 0.080 |
| **Cancer** | 0.42 (0.10 - 1.73) | 0.230 |  |  |  |
| **CIED implantation** |  |  |  |  |  |
| None | Reference |  |  |  |  |
| Any CIED implantation | 0.53 (0.30 - 0.95) | **0.034** | **-0.077** | 0.70 (0.38 - 1.27) | 0.238 |
| **Clinical parameters** |  |  |  |  |  |
| **Duration from diagnosis to improvement (per 1 y)** | 0.92 (0.85 - 1.00) | **0.049** | **-0.002** | 0.94 (0.86 - 1.02) | 0.136 |
| **NYHA class** |  |  |  |  |  |
| NYHA class I | Reference |  |  |  |  |
| NYHA class II/III/IV | 0.34 (0.16 - 0.70) | **0.003** | **-0.336** | 0.45 (0.20 - 0.91) | **0.026** |
| **Heart rate (per 10 bpm)** | 0.86 (0.67 - 1.11) | 0.252 |  |  |  |
| **Systolic blood pressure (per 1 mmHg)** | 1.00 (0.99 - 1.02) | 0.903 |  |  |  |
| **Diastolic blood pressure (per 1 mmHg)** | 1.01 (0.98 - 1.04) | 0.476 |  |  |  |
| **QRS duration (per 1 ms)** | 0.99 (0.98 - 1.00) | 0.052 |  |  |  |
| **Laboratory parameters** |  |  |  |  |  |
| **Glucose (per 1 mmol/L)** | 0.91 (0.74 - 1.10) | 0.331 |  |  |  |
| **eGFR (per 10 mL/min/1.73m²)** | 1.14 (1.01 - 1.28) | **0.034** | — |  |  |
| **Urea (per 1 mmol/L)** | 0.87 (0.77 - 0.98) | **0.020** | — |  |  |
| **Sodium (per 1 mmol/L)** | 1.05 (0.92 - 1.19) | 0.450 |  |  |  |
| **Potassium (per 1 mmol/L)** | 1.25 (0.64 - 2.46) | 0.510 |  |  |  |
| **AST (per 10 U/L)** | 0.86 (0.65 - 1.14) | 0.302 |  |  |  |
| **ALT (per 10 U/L)** | 0.95 (0.79 - 1.14) | 0.559 |  |  |  |
| **NT-proBNP (per 10 pmol/L)** | 0.90 (0.82 - 0.99) | **0.033** | — |  |  |
| **Hemoglobin (per 1 mmol/L)** | 0.87 (0.67 - 1.14) | 0.314 |  |  |  |
| **Imaging parameters** |  |  |  |  |  |
| **LA diameter (per 1 mm)** | 0.97 (0.93 - 1.01) | 0.118 |  |  |  |
| **LVEDD (per 1 mm)** | 0.95 (0.91 - 0.99) | **0.028** | **-0.013** | 0.95 (0.91 - 0.99) | **0.017** |
| **LVEF (per 1 %)** | 1.05 (1.00 - 1.10) | 0.070 |  |  |  |
| **VCI diameter (per 1 mm)** | 1.01 (0.94 - 1.08) | 0.873 |  |  |  |
| **TAPSE (per 1 mm)** | 1.03 (0.97 - 1.09) | 0.389 |  |  |  |
| **E/e’ ratio (per 1 unit)** | 0.99 (0.92 - 1.06) | 0.722 |  |  |  |
| **RV dysfunction** | 0.70 (0.31 - 1.56) | 0.379 |  |  |  |

Dual-drug therapy refers to a strategy in which patients either successfully discontinued MRA, loop diuretics, and digoxin or did not use these drugs for the entire duration, dependent on RASi and β-blockers alone. Demographic variables and other statistically significant variables in the univariable Cox regression analysis underwent LASSO regression. Only variables with non-zero coefficients were included in the multivariable Cox regression analysis. The dash symbol indicates variables excluded by LASSO regression due to coefficient shrinkage to zero.

ALT, alanine aminotransferase; AST, aspartate aminotransferase; CI, confidence interval; CIED, cardiac implantable electronic devices; eGFR, estimated glomerular filtration rate; HF, heart failure; HR, hazard ratio; ICM, ischemic cardiomyopathy; LA, left atrium; LASSO, least absolute shrinkage and selection operator; LVEDD, left ventricular end-diastolic diameter; LVEF, left ventricular ejection fraction; MRA, mineralocorticoid receptor antagonists; NICM, non-ischemic cardiomyopathy; NT-proBNP, N-terminal pro-b-type natriuretic peptide; NYHA, New York Heart Association; RASi, renin-angiotensin system inhibitors; RV, right ventricle; TAPSE, tricuspid annular plane systolic excursion; VCI, vena cava inferior.

**Supplementary Table 7** Cox regression analysis of dual-drug therapy

| **Variables (at the time of LVEF improvement)** | **Univariable Cox regression** | | **Multivariable Cox regression** | |
| --- | --- | --- | --- | --- |
|  | **Unadjusted HR (95% CI)** | ***P* value** | **Adjusted HR (95% CI)** | ***P* value** |
| Age (per 1 y) | 0.97 (0.95 - 0.99) | **0.005** | 0.99 (0.97 - 1.01) | 0.353 |
| Diabetes mellitus | 0.10 (0.01 - 0.70) | **0.013** | 0.17 (0.00 - 1.27) | 0.099 |
| Chronic kidney disease | 0.20 (0.06 - 0.63) | **0.006** | 0.40 (0.10 - 1.10) | 0.080 |
| Any CIED implantation | 0.53 (0.30 - 0.95) | **0.034** | 0.70 (0.38 - 1.27) | 0.238 |
| Duration from diagnosis to improvement (per 1 y) | 0.92 (0.85 - 1.00) | **0.049** | 0.94 (0.86 - 1.02) | 0.136 |
| NYHA class II/III/IV^a^ | 0.34 (0.16 - 0.70) | **0.003** | 0.45 (0.20 - 0.91) | **0.026** |
| LVEDD (per 1 mm) | 0.95 (0.91 - 0.99) | **0.028** | 0.95 (0.91 - 0.99) | **0.017** |

Dual-drug therapy refers to a strategy in which patients either successfully discontinued MRA, loop diuretics, and digoxin or did not use these drugs for the entire duration, dependent on RASi and β-blockers alone.

^a^ NYHA class I served as the reference category for NYHA class II/III/IV.

CI, confidence interval; CIED, cardiac implantable electronic devices; HR, hazard ratio; LVEDD, left ventricular end-diastolic diameter; LVEF, left ventricular ejection fraction; MRA, mineralocorticoid receptor antagonists; NT-proBNP, N-terminal pro-b-type natriuretic peptide; NYHA, New York Heart Association; RASi, renin-angiotensin system inhibitors.
